# Health resource allocation within the close-knit medical consortium after the Luohu healthcare reform in China: efficiency, productivity, and influencing factors

**DOI:** 10.3389/fpubh.2024.1395633

**Published:** 2024-08-29

**Authors:** Fangfang Gong, Ying Zhou, Junxia Luo, Guangyu Hu, Hanqun Lin

**Affiliations:** ^1^Department of Hospital Group Office, Shenzhen Luohu Hospital Group Luohu People’s Hospital, (The Third Affiliated Hospital of Shenzhen University), Shenzhen, China; ^2^Institute of Medical Information, Center for Health Policy and Management, Chinese Academy of Medical Sciences and Peking Union Medical College, Beijing, China

**Keywords:** health resource allocation, medical consortium, reform, productivity, efficiency

## Abstract

**Objective:**

This study aims to assess the efficiency and productivity of the Luohu Hospital Group after the reform and to identify factors influencing the efficiency to support the future development of medical consortia.

**Methods:**

Data on health resources from Shenzhen and the Luohu Hospital Group for the years 2015 to 2021 were analyzed using the super-efficiency slack-based measure data envelopment analysis (SE-SBM-DEA) model, Malmquist productivity index (MPI), and Tobit regression to evaluate changes in efficiency and productivity and to identify determinants of efficiency post-reform.

**Results:**

After the reform, the efficiency of health resource allocation within the Luohu Hospital Group improved by 33.87%. Community health centers (CHCs) within the group had an average efficiency score of 1.046. Moreover, the Luohu Hospital Group’s average total factor productivity change (TFPCH) increased by 2.5%, primarily due to gains in technical efficiency change (EFFCH), which offset declines in technical progress change (TECHCH). The efficiency scores of CHCs were notably affected by the ratio of general practitioners (GPs) to health technicians and the availability of home hospital beds.

**Conclusion:**

The reform in the Luohu healthcare system has shown preliminary success, but continuous monitoring is necessary. Future strategies should focus on strengthening technological innovation, training GPs, and implementing the home hospital bed policy. These efforts will optimize the efficiency of health resource allocation and support the integration and development of resources within the medical consortium.

## 1 Introduction

With advancements in society and economy and the accelerated population aging, health costs are rising in all countries. The World Health Organization announced that global spending on health more than doubled in real terms over the past two decades, reaching US$ 8.5 trillion in 2019, or 9.8% of global gross domestic product. Primary healthcare spending accounted for more than half of total health spending and amounted to an average of 3.1% of the gross domestic product (1). However, health resources are often wasted due to medical inefficiency (2). The World Health Organization believes that each country should follow economic principles to improve the efficiency of health resource allocation (3). Currently, improving efficiency has been identified as one of the four primary goals of the health system (4, 5) and is gradually becoming a central goal of health system development (6). In China, there has long been a structural imbalance in the allocation of healthcare resources, leading to an inverted triangle pattern and significant waste of health resources. To address these issues, the State Council of the People’s Republic of China issued the Outline of the National Medical and Health Care Service System Plan (2015–2020) in 2015 (7). This plan proposed optimizing the allocation of health resources and constructing an integrated system with comprehensive services, distinct divisions of labor, complementary functions, and close collaboration.

Luohu district of Shenzhen serves as a pilot area for China’s medical system reform. On August 20, 2015, the Luohu Hospital Group was officially established as a pilot urban medical consortium in China (8). The group, consisting of five hospitals and 45 community health centers (CHCs), serves over 4 million patients annually, primarily catering to the residents of the Guangdong-Hong Kong-Macao Greater Bay Area. The primary health issues of the population include chronic diseases such as diabetes, hypertension, and cardiovascular diseases. The group is active in preventive healthcare, promoting health education and disease prevention, aiming to improve the population’s overall health and reduce the incidence of diseases and hospitalization rates. The Luohu healthcare reform focuses on the health of residents, aiming to achieve a transformation from a “disease-centered” to a “people-centered” approach (9). Some scholars believe that a typical example of urban medical consortium construction in China is the Shenzhen Luohu model (10, 11). Over the past 8 years, Luohu’s healthcare reform had positive results and has been replicated in various parts of China.

Developed countries have evaluated the implementation of integrated healthcare services in different contexts. Exley et al. (12) performed semi-structured interviews with stakeholders in Italy, the Netherlands, and Scotland, discovering that engaging local leaders was crucial for achieving the integration agenda. Busse et al. (13) analyzed the outcomes of integrated care in Germany, the Netherlands, and England, identifying improvements in intermediate clinical outcomes, process metrics, and provider satisfaction. Bainbridge et al. (14) made an important attempt to empirically and comprehensively examine the integration of network-integrated palliative care in Brazil. Liaw et al. (15) analyzed and compared data from integrated primary care centers in Australia, and found that improved informatization through resource integration promotes primary care capacity. Developed countries implemented integrated healthcare early and conducted comprehensive evaluations. However, the construction of integrated healthcare services in China began late and was presented as urban medical consortia and county medical communities. Zhao et al. (16) found that China’s integrated healthcare delivery system enhanced primary care provider capacity, resource allocation, patient satisfaction, and primary care accessibility in pilot cities through the use of institutional data, in-person interviews, and expert consultations. Yuan et al. (17) evaluated the impact of vertical integration reforms on primary care facilities in China, finding that they significantly strengthened inpatient services and improved the quality of care for hypertension and diabetes in primary healthcare. Zhang et al. (18) conducted semi-structured interviews with 30 healthcare providers to investigate whether county medical communities could improve rural healthcare service administrators’ satisfaction with the vertical integration of the healthcare system. The study found that to promote county medical communities, administrators of key county hospitals should adopt healthcare system integration strategies. Liang et al. (9) conducted semi-structured interviews and focus groups with key informants in the Chinese healthcare consortium. The study found that the reforms strengthened the coordination of primary care and improved the quality and efficiency of medical services. Some literature has examined the impact of integrated healthcare system reforms but mainly focuses on the effects of reforms on medical staff, patient satisfaction, and the quality of care. There are fewer empirical studies on the efficiency of integrated healthcare services, particularly quantitative studies based on statistical modeling.

The Outline of the Healthy China 2030 Plan proposes that by 2050, China will have established a healthy nation that is compatible with its socialist modernization (19). The problem of irrational health resource allocation still exists in China, and optimizing the allocation of health resources is crucial to achieving the “Healthy China” strategy. As a pilot project in the integrated medical service system, it is important to understand the efficiency of the Luohu medical consortium for further healthcare reform. The main objectives of this study are: (1) To investigate the trends of configurational efficiency and productivity changes of the Luohu Hospital Group during the healthcare reform in Luohu from 2015 to 2021, considering horizontal and vertical perspectives, respectively. (2) To identify the factors affecting the efficiency of regional and CHCs, based on efficiency measurements. (3) To provide a scientific basis and feasible solutions for constructing medical consortia around the world, and to help maximize the efficiency of medical resource allocation within the medical consortium.

The remainder of the paper is organized as follows: Section 2 details data acquisition and methodological choices. Section 3 presents the results of the analysis of relative efficiency, productivity, and influencing factors in 2015–2021. Section 4 discusses the findings and policy recommendations. Section 5 outlines the strengths and limitations of this study. Finally, Section 6 provides a summary of the study.

## 2 Methods

### 2.1 Data sources

**Figure 1:**
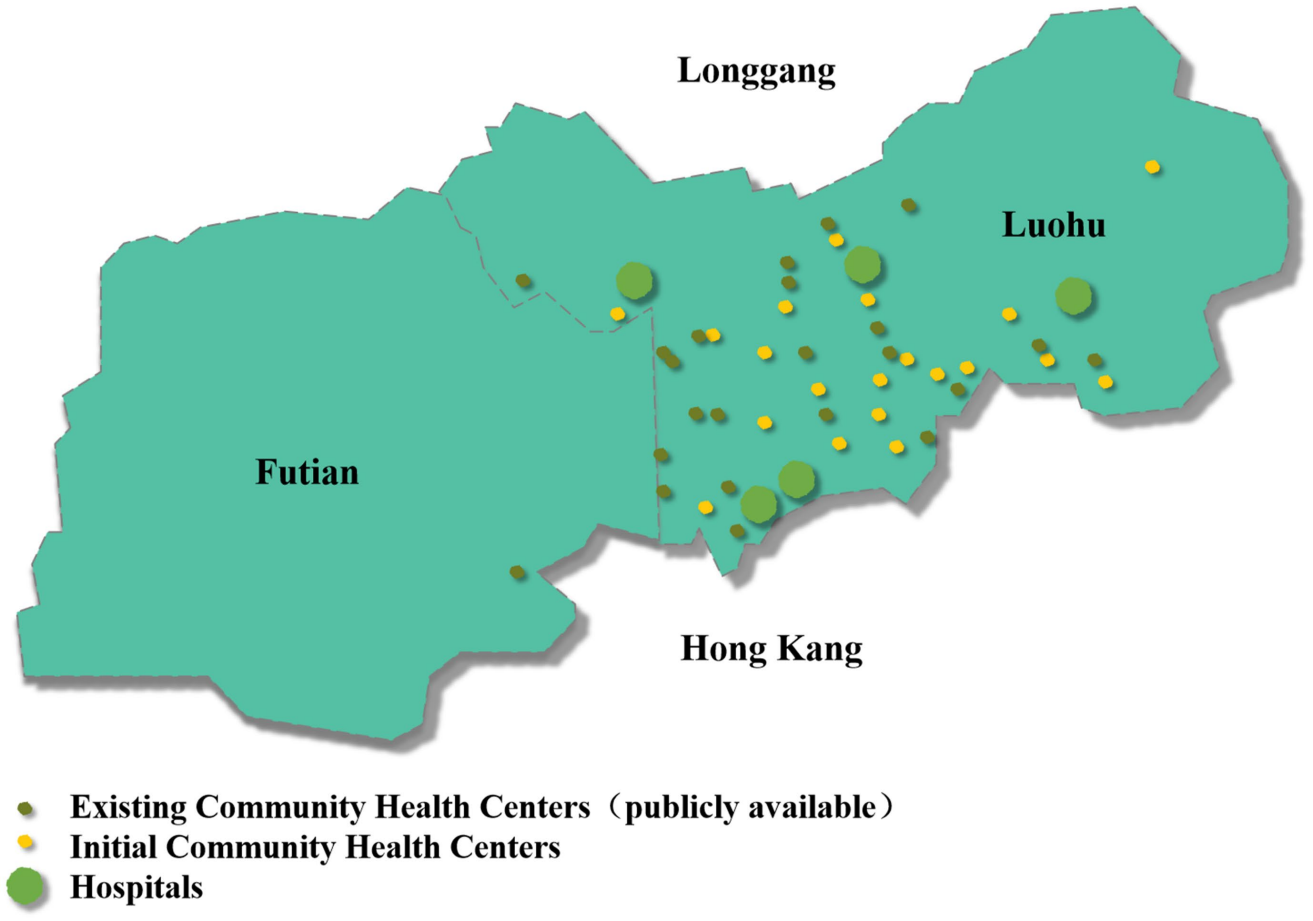
Distribution of CHCs and hospitals within the Luohu Hospital Group.

This study collected health resource data from 2015 to 2021, extracted from the information system of the Luohu Hospital Group and the statistical yearbooks of Shenzhen districts (Futian Statistical Yearbook, Luohu Statistical Yearbook, Yantian Statistical Yearbook, Nanshan Statistical Yearbook, Bao’an Statistical Yearbook, Longhua Statistical Yearbook, Pingshan Statistical Yearbook, Guangming Statistical Yearbook, and Dapeng New District Statistical Yearbook). Due to missing data, Longgang District was not included. Consequently, the nine districts mentioned above and the Luohu Hospital Group were selected as the 10 decision-making units (DMUs) for evaluating the relative efficiency of regional health resources. Twenty CHCs, all with a floor area of 90 m^2^ or more and included from the beginning of the establishment of the Luohu Hospital Group, were selected as DMUs (Figure 1) for the internal efficiency evaluation of the Luohu Hospital Group.

### 2.2 Variables selection

We selected input and output indicators with reference to past studies (20–25). To ensure the credibility of the results, the number of DMUs was greater than twice the total of input and output indicators (26–28). Input indicators for regional health resources included health workers (I1) and beds (I2); output indicators included outpatient and emergency visits (O1) and hospital discharges (O2). Input indicators for community health centers included health workers (I3), floor area (I4), equipment (I5), and home hospital beds (I6). Output indicators included the number of outpatient visits (O3), medical services in home hospital beds (O4), health education (O5), preventive medical services (O6), and chronic disease management (O7). The number of beds was not selected as an input indicator because there no beds were allocated within the CHCs of the Luohu Hospital Group. Table 1 explains the meaning of the selected indicators. The regions evaluated in this study all have a three-level healthcare structure and offer similar services. The CHCs offer the same service content and are all public healthcare organizations under the jurisdiction of the Luohu Hospital Group. They all have the same input and output indicators in absolute numbers, making them homogeneous and comparable.

**Table 1:** Definition of the selected variable.

Type	Variable	Definition
Regional health resources	*Inputs*	
	Health workers	Total number of health workers (doctor, nurse, pharmacist, and medical technician)
	Bed.	The number of hospital beds
	*Outputs*	
	Visits	Total number of outpatient and emergency visits
	hospital discharges	The number of people discharged after hospitalization
Community health centers	*Inputs*	
	Health workers	Total number of health workers (general practitioners, general nurses, community clinical pharmacists, public health physicians, TCM physicians, and medical technicians)
	Floor area	Total calculation of the floor area of each floor
	Equipment	The number of devices with a value exceeding CNY 5000
	Home hospital beds	The number of home hospital beds set up throughout the year
	*Outputs*	
	Outpatient visits	Traditional Chinese medicine and Western medicine
	Number of home bed medical services	Total number of medical services carried out through family hospital beds
	Health education	The number of health education and publicity sessions conducted
	Preventive medical services	Total number of breast cancer cervical cancer lung cancer screenings, vaccinations
	Chronic disease management	The number of people managed for chronic diseases

### 2.3 DEA and Malmquist index

Data Envelopment Analysis (DEA) has been widely used to evaluate the efficiency of hospitals in many countries, such as China (29–33), England (34), Japan (35), Turkey (36), etc. (37). DEA is a performance evaluation method developed in 1978 by Charnes et al., renowned American operations researchers, based on Farrell’s seminal exposition of efficiency (38). This method does not require the estimation of a production function. The efficiency of multiple DMUs with multiple inputs and outputs at different periods can be evaluated by selecting input and output indicators. It is important to clarify that efficiency in DEA represents relative efficiency. Relative efficiency means that each DMU’s performance is assessed in comparison to others in the dataset, rather than against an absolute standard. A DMU is considered efficient if it lies on the efficiency frontier, constructed from the best-performing units achieving the maximum possible outputs for given inputs or the minimum inputs for given outputs. When new units are added, the efficiency frontier may change, and some units may become inefficient.

Commonly used models for the static efficiency studies in DEA include the Charnes-Cooper-Rhodes (CCR) model (39) and the Banker-Charnes-Cooper (BCC) model (40). The CCR model assumes constant returns to scale, and the calculated technical efficiency incorporates scale efficiency, referred to as integrated technical efficiency. Kirigia et al. (35) used the CCR model to analyze the efficiency of community hospitals in Eritrea and found that only 42% of hospitals achieved scale efficiency. Li et al. (32) used the CCR model and found that the relative efficiency of county-level public hospitals in Anhui Province, China, was trending downward. County-level public hospitals need to assess their realities and identify their deficiencies. The BCC model excludes the effects of scale efficiency. Technical efficiency is further divided into pure technical efficiency and scale efficiency components for consideration. This model is suitable for contexts with variable returns to scale and is highly adaptable to the varying conditions of real-life production. Wang et al. (31) used the BCC model to analyze the efficiency of county-level maternal and child health centers in rural areas of Guangxi, China. The study indicated that the overall operational efficiency of hospitals is low and needs improvement. For hospitals in impoverished areas, policymakers should consider not only investing in hardware facilities but also introducing advanced technology and high-level medical staff to enhance technical efficiency. However, this traditional radial model does not include slack variables for inefficiency measurement, which can lead to errors in efficiency measurement. Tone (41) fully considered input and output slacks and proposed a non-radial DEA model, the slack-based measure (SBM) model (19), which addresses this issue effectively. Nevertheless, this model cannot further compare DMUs deemed efficient. Building on this model, Tone proposed the super-efficiency slack-based measure (SE-SBM) model (42). This model allows the efficiency value of effective DMUs to exceed 1, enabling comparison among different effective DMUs and aiding in identifying the best ones (43–45). The SE-SBM model was applied to measure the efficiency of healthcare services in district-level public general hospitals. When *ρ* = 1, the DMU is evaluated as SE-SBM efficient. The following is the formula for the SE-SBM model (Equation 1):

(1) $$\begin{array}{c} \mathrm{minimize}\;p=\frac{\frac{1}{m}\sum_{i=1}^{n}\frac{{\bar{x}}_{ik}}{x_{ik}}}{\frac{1}{q}\sum_{r=1}^{q}\frac{{\bar{y}}_{rk}}{y_{rk}}}\\ \mathrm{subject}\;to\;\overset{n}{\underset{j=1}{\sum }}x_{ij}\lambda_{j}\leq {\bar{x}}_{ik},\overset{n}{\underset{j=1}{\sum }}y_{rj}\lambda_{j}\geq {\bar{y}}_{rk},x_{ik}\leq \\ {\bar{x}}_{ik},y_{ik}\geq {\bar{y}}_{ik},\lambda_{j},{\bar{y}}_{rk}\geq 0 \end{array}$$

where *m* and *q* represent the input and output variables of the DMU, respectively; *x*_ik_ and *y*_rk_ denote the i_th_ input and r_th_ output of the DMU, respectively. *λ* is the adjustment matrix. *ρ* takes a value in the range of 0 to 1. When *ρ* = 1, it indicates that the DMU is more efficient and is at the efficiency frontier, and the respective slacks are 0. When it is close to 0, it indicates that the DMU is inefficient.

The Malmquist index is a longitudinal comparative analysis of input and output efficiencies using the DEA model to measure the Malmquist total factor productivity index (46). It is usually used in dynamic efficiency studies to assess changes in productivity, which can be referred to as dynamic efficiency, reflecting efficiency changes over time. It is expressed by the following formula (47) (Equation 2):

(2) $$\begin{array}{c} \;{M_{i}}^{t+1}\left(Y^{t+1},X^{t+1},Y^{t},X^{t}\right)=\frac{D^{t+1}\left(Y^{t+1},X^{t+1}\right)}{D^{t}\left(Y^{t},X^{t}\right)}\\ \times {\left[\frac{D^{t}\left(Y^{t+1},X^{t+1}\right)}{D^{t+1}\left(Y^{t+1},X^{t+1}\right)}\times \frac{D^{t}\left(Y^{t},X^{t}\right)}{D^{t+1}\left(Y^{t},X^{t}\right)}\right]}^{\frac{1}{2}}\; \end{array}$$

MPI, also known as total factor productivity change (TFPCH), is an intertemporal evaluation of productivity that addresses the limitations of radial DEA models in analyzing efficiency dynamically. TFPCH reflects the change in total factor productivity between t and t + 1. TFPCH >1 indicates an increase in productivity. TFPCH <1 indicates a decrease, and TFPCH = 1 is stagnant.

TFPCH can be decomposed into technical efficiency change (EFFCH) and technical progress change (TECHCH) components (48). EFFCH indicates the extent to which a decision unit moves closer to or farther from the production frontier between periods t and t + 1 and can be further decomposed into pure technical efficiency change (PECH) and scale efficiency change (SECH). The Equation 3 is expressed as follows:

(3) $$\mathrm{TFPCH}=\mathrm{EFFCH}\times \mathrm{TECHCH}=\left(\mathrm{PECH}\times \mathrm{SECH}\;\right)\times \mathrm{TECHCH}$$

If EFFCH >1, it indicates that technical efficiency is close to the production frontier. If EFFCH = 1, it indicates that the technical efficiency is unchanged. If EFFCH <1, it indicates a decrease in technical efficiency. TECHCH indicates a change in the efficiency frontier. If TECHCH >1, the technical frontier advances to a higher level. If TECHCH = 1, the technical frontier remains unchanged. If TECHCH <1, the technology frontier recedes.

### 2.4 Tobit regression

Tobit regression models apply to situations where the dependent variable is truncated (49) and are commonly used to analyze the factors influencing the efficiency of DEA in healthcare organizations (45, 50). In this study, the factors influencing the efficiency of regional health resource allocation were analyzed using the value of regional health resource allocation super-efficiency as the dependent variable, and the *per capita* gross domestic product (PGDP), population size, and health literacy level as the independent variables. Factors influencing the efficiency of CHCs were analyzed using the super-efficiency value of CHCs as the dependent variable, and the percentage of general practitioners (GPs) to health technicians, the floor area, the number of home hospital beds, and the number of pieces of equipment costing more than 5,000 yuan as independent variables.

### 2.5 Data analysis

Data entry and descriptive statistics of input–output indicators were performed using Microsoft Excel 2016. DEAP 2.1 and Dearun 3.1 were used to analyze the operational efficiency of hospitals. STATA 17.0 was applied to further analyze the factors influencing the efficiency of the regional health resource allocation and CHCs using the Tobit regression model.

## 3 Results

### 3.1 Descriptive analysis

From 2015 to 2021, the health resources of the Luohu Hospital Group increased significantly. As shown in Tables 2, 3, the input indicators, including the number of health workers and the number of beds in the group, showed an upward trend from 2015 to 2021. For the output indicators, the number of visits and discharges showed an upward trend from 2015 to 2019, the number of visits and discharges decreased from 2019 to 2020 and resumed rising in 2021. The fastest-growing input indicators for the community were the number of equipment over 5,000 yuan and the number of home hospital beds. The fastest-growing output indicators were the number of preventive medical services and the number of patients managed for chronic diseases.

**Table 2:** Descriptive statistics for input variables.

Input					
Luohu Hospital Group		CHCs			
I1^a^	I2^b^	I3^a^	I4 (m^2^)	I5	I6^b^
2,465	1,010	331	12,984	324	508
2,578	1,068	348	12,984	422	683
2,831	1,109	394	14,184	643	810
3,313	1,479	458	16,866	872	668
3,849	1,839	474	19,190	1,102	604
4,048	1,890	531	20,385	1,358	660
4,376	1,890	553	20,985	1,381	883

a Person.

b Bed.

**Table 3:** Descriptive statistics for output variables.

Output						
Luohu Hospital Group		CHCs				
O1^c^	O2^a^	O3^c^	O4^d^	O5^a^	O6^d^	O7^a^
2,455,737	35,472	953,455	388	175,766	3,038	4,050
2,883,925	41,868	1,436,146	423	192,089	2,773	7,962
3,344,179	45,109	1,441,371	532	225,676	3,220	9,287
3,616,063	50,554	1,236,096	497	234,557	3,509	15,071
4,084,031	64,501	1,515,986	707	249,084	4,042	19,496
3,426,196	56,451	1,012,291	531	278,740	3,986	23,735
4,879,238	66,404	1,141,298	603	1,687,564	6,330	28,465

a Person.

c Person.time.

d Frequency.

### 3.2 Analysis of efficiency and productivity level of the Luohu Hospital Group

**Figure 2:**
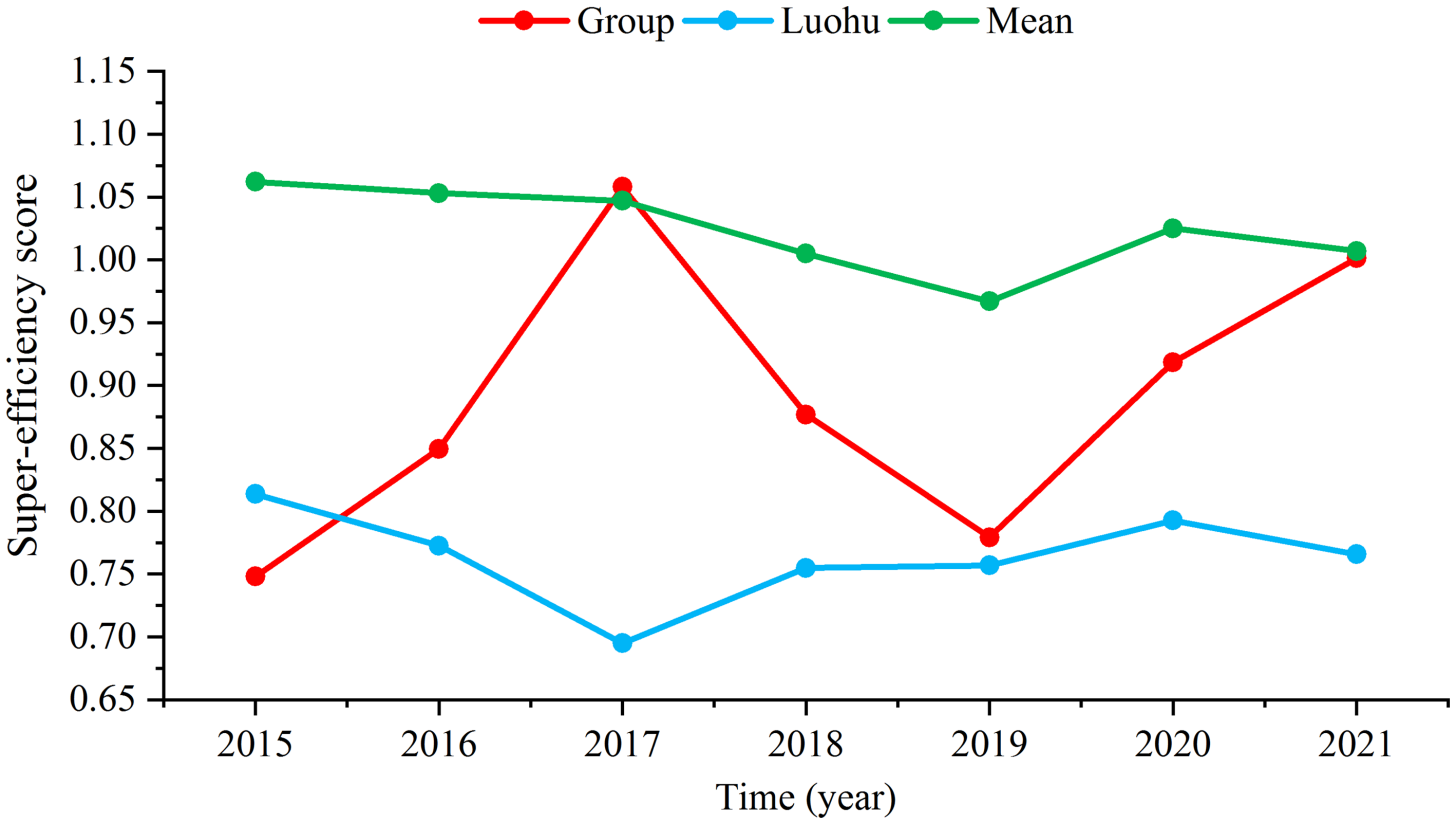
Super-efficiency scores for regional health resources in 2015–2021.

As shown in Supplementary Table S1, the Luohu Hospital Group has an average super-efficiency value of 0.890 and achieved DEA efficiency in 2017 (score = 1.058) and 2021 (score = 1.001). The overall trend of the group’s super-efficiency has been upward, surpassing the efficiency level of Luohu district since 2016 (Figure 2). The group experienced the largest increase from 2015 to 2021, with a super-efficiency growth rate of 33.87% (Supplementary Figure S1). The MPI was used to analyze the productivity changes from 2015 to 2021, as shown in Table 4. The average TFPCH of the Luohu Hospital Group was 1.020 in 2015–2021, rising by 2.0%. Decomposing the TFPCH further reveals that the reason for this trend is that the EFFCH rises by 6.8% while the TECHCH declines by 3.1%. The decline in TECHCH is common and occurs in other Shenzhen regions. The average total factor productivity of health resources in the Shenzhen region in this study was 0.990, a decline of 1.0%, mainly due to the decline in TECHCH.

**Table 4:** Productivity change by regions during 2015–2021.

Regions	EFFCH	TECHCH	PECH	SECH	TFPCH	Mean
Luohu Hospital Group	1.068	0.969	1.061	1.007	1.020	1.025
Nanshan	1.092	0.946	1.040	1.075	1.013	1.033
Futian	1.016	0.985	0.997	1.024	0.999	1.004
Baoan	1.059	0.964	1.009	1.050	1.015	1.020
Longhua	0.947	0.999	0.937	1.009	0.926	0.964
Yantian	1.027	0.977	1.010	1.019	1.006	1.008
Pingshan	0.922	0.989	0.925	1.013	0.892	0.948
Guangming	1.038	0.961	1.020	1.016	1.009	1.009
Dapeng	1.070	0.977	1.016	1.060	1.039	1.033
Luohu	1.022	0.973	0.992	1.030	0.985	1.000
Mean	1.026	0.974	1.001	1.030	0.990	1.004

EFFCH, technical efficiency change; TECHCH, technical progress index change; PECH, pure technical efficiency change; SECH, scale efficiency change; TFPCH, total factor productivity change.

### 3.3 Analysis of efficiency and productivity levels of CHCs within the group

**Figure 3:**
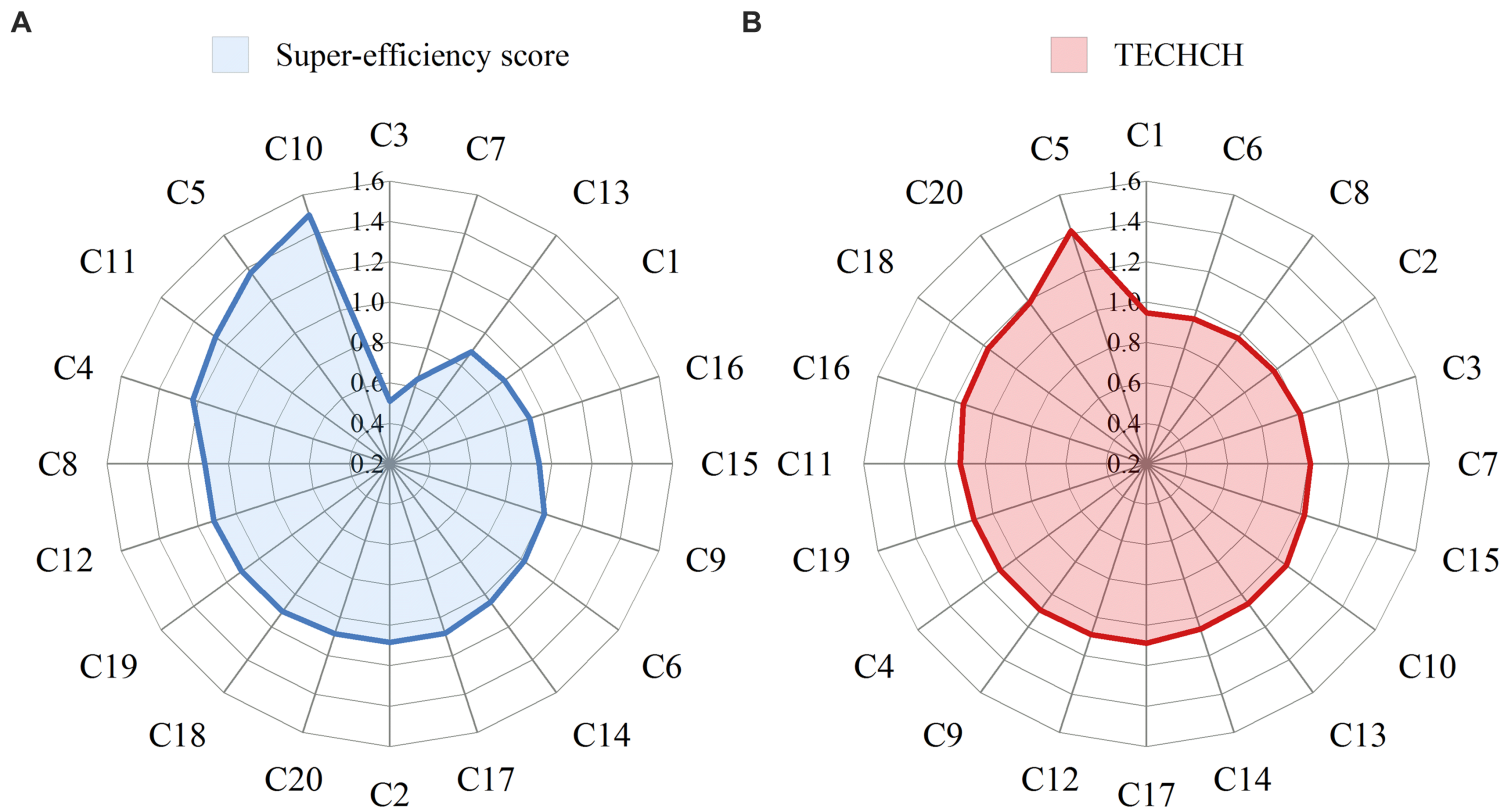
The average of the super-efficiency score **(A)** and TECHCH **(B)** for the 20 CHCs.

**Figure 4:**
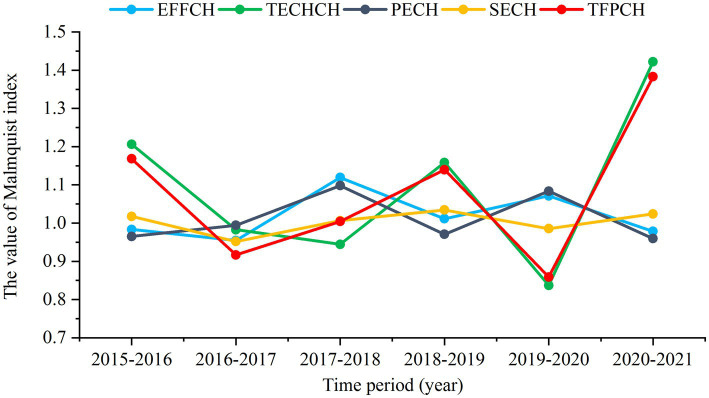
Productivity change by CHCs during 2015–2021.

As shown in Supplementary Tables S2, S3, the mean value of super-efficiency for CHCs in the group was 1.046, and all years were DEA efficient except for 2017 (score = 0.987). The number of CHCs that achieved DEA efficiency from 2015 to 2021 was 17, 16, 15, 17, 15, 19, and 15, respectively. The CHC with the highest super-efficiency was C10 (score = 1.494), followed by C5 (score = 1.370) and C11 (score = 1.267) (Figure 3A). The average TFPCH for CHCs in 2015–2021 was 1.078, with TFPCH increasing during 2015–2016, 2017–2018, 2018–2019, and 2020–2021, and trending downward in 2016–2017 and 2019–2020 (Figure 4). The reasons for the decline in these two periods were different. The decline in 2016–2017 was affected by EFFCH and TECHCH, but the effect of EFFCH was greater than that of TECHCH. Further analysis of the decline in PECH and SECH by 0.6 and 4.8%, respectively, revealed that the decline in EFFCH was mainly affected by SECH. The decline in 2019–2020 was influenced by TECHCH, which declined by 16.3%. Table 5 shows the results of the MPI analysis for the 20 CHCs in 2015–2021, where 15 CHCs showed an upward trend in productivity. The CHC with the highest rise in TFPCH was C5 (score = 1.412), followed by C20 (score = 1.185) and C18 (score = 1.170) (Figure 3B). The remaining 5 CHCs showed a decline ranging from 0.2 to 5.3%.

**Table 5:** Productivity change by CHCs during 2015–2021.

CHCs	EFFCH	TECHCH	PECH	SECH	TFPCH
C1	0.971	1.050	1.020	0.937	0.947
C2	1.000	0.979	1.000	1.000	0.979
C3	0.953	1.062	0.956	0.997	0.998
C4	1.140	1.052	1.064	1.013	1.097
C5	1.000	1.412	1.000	1.000	1.412
C6	0.968	0.988	0.961	1.029	0.955
C7	1.024	1.079	1.013	1.006	1.010
C8	0.965	1.011	1.000	0.965	0.969
C9	1.044	1.125	1.023	1.003	1.095
C10	1.000	1.055	1.000	1.000	1.055
C11	1.000	1.122	1.000	1.000	1.122
C12	1.000	1.089	1.000	1.000	1.089
C13	1.042	1.144	1.022	1.000	1.057
C14	0.976	1.067	1.007	0.979	1.062
C15	1.022	1.057	1.036	0.983	1.022
C16	1.137	1.048	1.134	1.004	1.155
C17	1.024	1.062	1.000	1.024	1.089
C18	1.064	1.112	1.000	1.064	1.170
C19	1.009	1.110	1.000	1.009	1.099
C20	1.053	1.213	1.000	1.053	1.185
Mean	1.020	1.092	1.012	1.003	1.078

### 3.4 Results of the Tobit regression model

The results are shown in Supplementary Table S4. PGDP, population size, and health literacy (*p* > 0.05) had no significant effects on the efficiency of regional health resource allocation. As shown in Supplementary Table S4, the percentage of GPs (*p* < 0.01) and the number of home hospital beds (*p* < 0.05) were positively related to CHCs efficiency, while floor area (*p* > 0.05) and the percentage of intermediate-level staff (*p* > 0.05) did not affect CHCs’ efficiency.

## 4 Discussion

The static analysis of regional resource allocation shows that after the reform began in 2015, the super efficiency score showed an upward trend, and DEA efficiency was not achieved until 2017. This problem may be caused by the lagging nature of the reform, a phenomenon observed in many studies (51, 52). The ‘valley’ of the efficiency values appeared in 2018 and 2019, which may be related to the relocation of hospitals in the group starting in 2018, resulting in a decline in the total number of consultations. Nevertheless, the overall trend in super-efficiency values for the Luohu Hospital Group is positive, with the group achieving efficiency again in 2021. This improvement signifies enhanced resource allocation after the reform, and the medical consortium has continuously optimized the rational allocation of medical resources through close cooperation among units. The MPI analysis further supports this result, showing TFPCH greater than 1, indicative of an upward trend in resource efficiency. However, a detailed analysis reveals that the increase in EFFCH conceals a decrease in TECHCH. Despite gains in PECH and SECH, the declining TECHCH poses challenges to the sustainable development of health resources. Notably, this study identifies a region-wide decline in TECHCH across Shenzhen, underscoring the critical need for advancing technological innovation and progress in healthcare.

The static efficiency analysis of CHCs revealed an average super-efficiency score of 1.046, indicating improved efficiency of CHCs after the reform. This improvement is attributed to the Luohu Hospital Group’s emphasis on grassroots healthcare, enhanced resource sharing within the medical consortium, and the strategic allocation of superior resources to the foundational healthcare level. The most efficient CHC is C10. Despite not being dominant in terms of medical resources, it is conveniently located within an apartment complex and has become the first line of defense for residents’ health. The current distribution of units within the Luohu Hospital Group is shown in Figure 1. The high efficiency proves the effectiveness of the primary healthcare reform by optimizing the layout of the CHCs, ‘encrypting’ the CHC service network, and constructing a ‘15-min primary healthcare service circle.’ The MPI analysis of social health centers revealed an upward trend in overall CHC productivity, but it identified two ‘valleys’ in productivity (Figure 4). The decline in total factor productivity in 2019–2020 was affected by COVID-19, which posed a great challenge to technological progress at this stage. Another downturn, attributed to reduced scale efficiencies in 2016–2017, coincides with a period of expansion for CHCs in 2017. This contrasts with the 2015 to 2016 period, which was marked by declines in technical efficiency due to a focus on scale expansion rather than on health technology and management improvements. Culyer (53) pointed out that if scale expansion exists in a healthcare organization, then the operational efficiency will decrease as the scale increases beyond the optimal size. This highlights the necessity for scientifically planned health resource investments and adjustments based on local needs.

The largest increase in TFPCH within the CHCs was C5, up 41.2%. C5, transformed from a hospital outpatient department, carries the earliest memories of many Luohu residents about medical care. This CHC has actively carried out reforms under the group’s leadership, serving as a microcosm of the Luohu reform. The CHC has explored multiple service models, forming a family doctor team with the ‘4 + X’ model. The ‘4’ represents one GP, one general nurse, one public health physician, and one community clinical pharmacist. The ‘X’ includes specialists, rehabilitation therapists, psychotherapists, social workers, and other relevant personnel who serve contracted residents. CHCs also vigorously promote the development of traditional Chinese medicine (TCM), having set up a TCM rehabilitation department and carrying out more than 20 TCM techniques, such as acupuncture and tuina. They advocate that the first diagnosis should be made at CHCs. If a referral is necessary, the doctor transfer the patient to the designated department through the group’s green channel. This ensures that the contracted residents are given priority in diagnosis and treatment and that the loop is closed for consultation and diagnosis.

Our previous study (54) found that the core strategies and mechanisms of the Luohu model enhance public health emergency response capacity, reflecting the advantages of integrated healthcare services. This study observes a similar situation, with 95% of CHCs achieving effective DEA in 2020, the highest percentage in 7 years. The dynamic analysis shows that technical efficiency rose by 7.1% from 2019 to 2020. It demonstrates that CHCs can fully integrate resources and achieve effective resource utilization during the coronavirus disease 2019 pandemic, effectively testing their management level in the face of acute events.

Despite the improvement in overall efficiency, the problem of balancing scale expansion and technological progress requires further optimization. Retaining patients in primary care is not only a crucial objective of healthcare reform but also an effective strategy for reducing the challenges and expenses associated with accessing healthcare for the public. However, relying solely on scale expansion to improve the primary care environment and attract patients is insufficient; technical investment must also be increased. A clear policy should be formulated to ensure that a fixed proportion of the budget for CHCs’ expansion is allocated to technology investment and medical personnel training. This will facilitate service expansion without neglecting technological advancement and the enhancement of medical staff capacity. A gradual expansion strategy should be adopted to promote primary care expansion in phases, with evaluations at the end of each phase to ensure newly established CHCs achieve sufficient efficiency before proceeding. In addition, leadership and organizational coordination should be strengthened to improve integration within the medical consortium. Effective leadership and organizational mechanisms are crucial for ensuring close cooperation and resource sharing among various units within the medical consortium, thereby enhancing overall operational efficiency and service quality.

There are varying perspectives on the effect of PGDP on efficiency. Some studies claim that PGDP has a positive effect on the efficiency of health resources (42, 55), while others argue that there is no effect (45, 56). The results of this study are consistent with the latter, and we found that PGDP had no significant effect on efficiency. It has been observed that research indicating a correlation typically utilizes national or provincial-level data, whereas those finding no correlation opt for county-level data within a province or district-level data within a city. Government policies and relatively small regional economic disparities may have weakened the impact. Leng et al. found that policy implementation narrowed the gap in health resource allocation between regions, largely benefiting from the guiding role of government policies, effectively improving health organizations’ service efficiency (57). Health reform policies significantly impact efficiency, weakening the economy’s influence. Additionally, the smaller variances in economic development within the same province or city, compared to those between different countries and regions, result in a less pronounced economic impact.

The results of Tobit regression of internal influencing factors on CHCs indicate that the percentage of GPs and the number of home hospital beds positively affect the efficiency of CHCs, and no significant effect was found for the percentage of floor area and intermediate-level staff. This finding is consistent with the direction of Luohu’s healthcare reform, where the group guides and supports GPs to work in primary health facilities by increasing salaries and expanding the social welfare for health workers. Luohu’s healthcare reform focuses on strengthening the development of primary healthcare personnel, particularly the training of GPs, and encourages specialist doctors to undergo transfer training in general medicine. Luohu Hospital Group, in conjunction with the Shenzhen Medical Continuing Education and Training Center, operates a one-year, full-time, off-the-job transfer training course for GPs. After completing the training, trainees participate in the unified examination of Guangdong Province. Those who qualify are issued a certificate of competency by the Guangdong Provincial Department of Health. Currently, the number of GPs in the group’s CHCs has increased from 156 before the reform to 672, a 331% increase. Additionally, Shenzhen has piloted the provision of home hospital beds for an extended period and has incorporated them into social security payments. The Luohu Hospital Group, as the main practicing unit, has vigorously developed home hospital beds since its founding, building a total of 7,834 beds through hospital outpatient inquiries, family members seeking help, and two-way referrals. These efforts reflect a trend toward prioritizing primary healthcare and community-based services, recognizing the essential role of GPs and integrated care home hospital beds in improving the healthcare system’s overall performance.

GPs are regarded as the gatekeepers of residents’ health. Establishing and refining a system of transfer training for GPs is an effective way to address the shortage of GPs and a crucial measure to strengthen the grassroots health workforce (51). We recommend enhancing the transfer training of GPs and ensuring their compensation. Organize diverse training formats and implement targeted training content for primary healthcare institutions. Establish an effective assessment and evaluation system to ensure the quality of GPs. Home hospital beds, an emerging health service, provide convenient and effective medical care for patients bedridden for long periods and who have difficulty going out (58). We recommend raising residents’ awareness of home hospital bed services, adapting these beds to the needs of an aging population, and increasing their number to better meet the needs of older adults and home care needs.

## 5 Strengths and limitations

This study comprehensively assessed the efficiency of the pilot medical consortium after health reform policy implementation, whereas existing studies typically focus on entire regions. However, the policy of integrating healthcare is primarily implemented within medical consortia. This study demonstrated the effects of healthcare reform policies on medical consortia both overall and internally, accumulating valuable experience for the future promotion of integrated healthcare. This study has several limitations. We compared the medical consortium as a region with other regions in Shenzhen. Although the structure of medical institutions within the medical consortium is consistent with that of the region as a whole, a comparative analysis with other similar medical consortia was lacking. Additionally, due to limited data access, we were unable to include more environmental variables in our analysis. In the future, it would be beneficial to compare with other similar medical consortia and incorporate more factors such as health condition, socioeconomic level, educational level, and the level of marginalization of localities into the analysis.

## 6 Conclusion

This paper combines the SE-SBM-DEA and Tobit models to analyze the relative efficiency, productivity, and influencing factors of the Luohu Hospital Group and CHCs after the reform from 2015 to 2021. Our findings indicate that, first, an overall increase in health resource allocation within the Luohu Hospital Group since the 2015 reforms, with efficiency and productivity experiencing fluctuations but ultimately showing an upward trend. These reforms have resulted in efficient resource allocation and productivity gains. Additionally, the integrated care model has proven beneficial for managing acute events, though it remains to be seen if these benefits are sustainable long-term. Secondly, it is important to recognize the impact of economies of scale and scientifically scale up inputs. There is also an urgent need to enhance technological innovation to support the sustainable development of health resources. Thirdly, the efficiency of medical resource allocation within CHCs is influenced by the proportion of the number of GPs and the number of home hospital beds. The cultivation of GPs and the implementation of the home hospital bed policy should be emphasized.

## Data Availability

The original contributions presented in the study are included in the article/Supplementary material, further inquiries can be directed to the corresponding author.
