# Supplementary material for: Health resource allocation within the close-knit medical consortium after the Luohu healthcare reform in China: efficiency, productivity, and influencing factors
Source: Front Public Health. 2024 Aug 29;12:1395633. doi: 10.3389/fpubh.2024.1395633 (PMC11390686; doi:10.3389/fpubh.2024.1395633)
Supplement: Supplementary file 1 [file Data_Sheet_1.docx]

***Supplementary Material***

**Supplementary Figures**


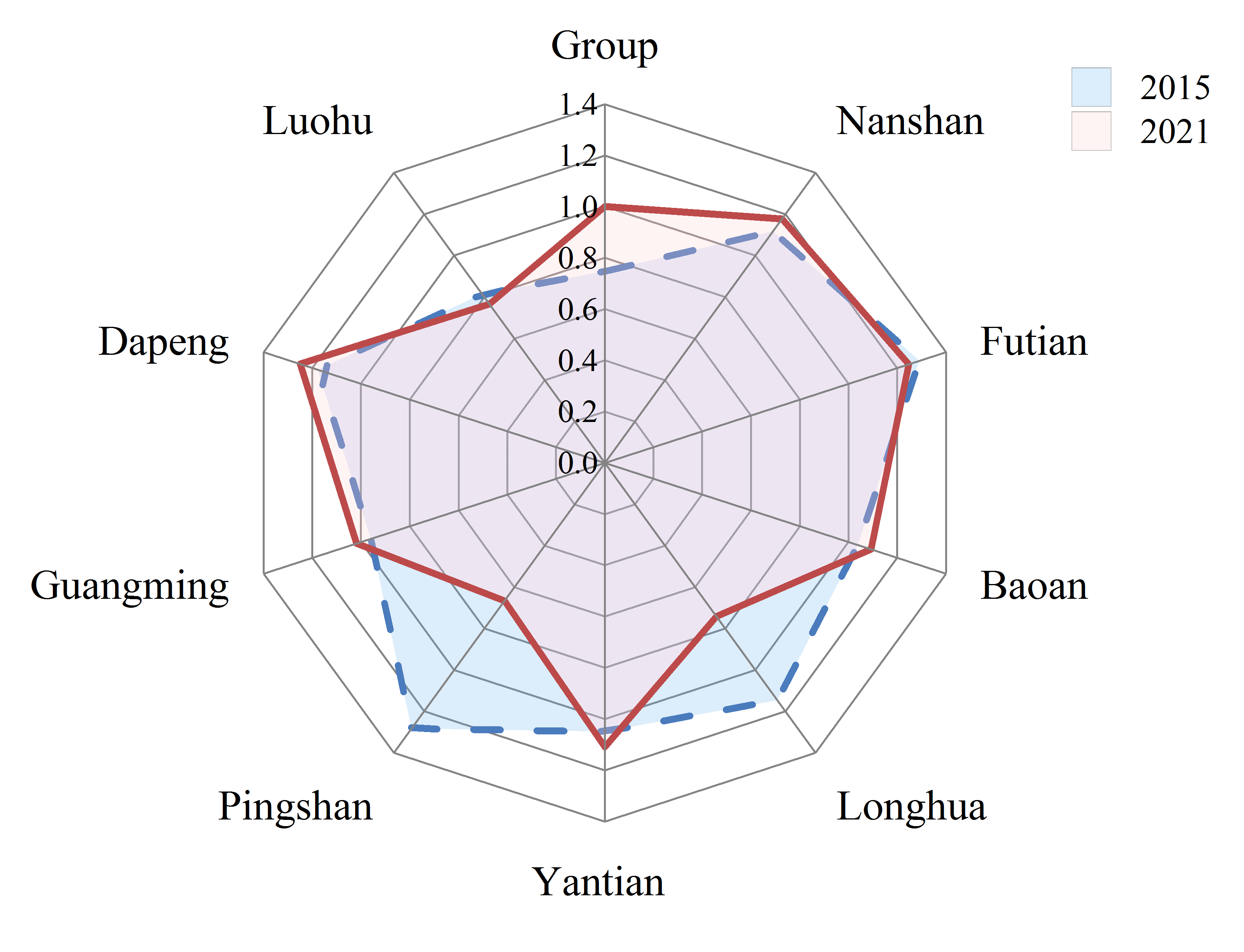


**FIGURE S1** Super-efficiency scores of regional health resources in 2015 and 2021.

**Supplementary Tables**

**TABLE S1** SE-SBM analysis of regional health resources in Shenzhen from 2015 to 2021.

| **Regions** | **2015** | **2016** | **2017** | **2018** | **2019** | **2020** | **2021** | **Mean** |
| --- | --- | --- | --- | --- | --- | --- | --- | --- |
| Luohu Hospital Group | 0.748 | 0.850 | 1.058 | 0.877 | 0.779 | 0.919 | 1.001 | 0.890 |
| Nanshan | 1.117 | 1.078 | 0.758 | 0.585 | 0.743 | 1.030 | 1.177 | 0.927 |
| Futian | 1.291 | 1.308 | 1.141 | 1.128 | 1.095 | 1.229 | 1.247 | 1.206 |
| Baoan | 1.039 | 1.034 | 1.061 | 1.041 | 1.057 | 1.052 | 1.093 | 1.054 |
| Longhua | 1.145 | 1.136 | 1.152 | 1.174 | 1.068 | 1.054 | 0.741 | 1.067 |
| Yantian | 1.048 | 1.030 | 1.043 | 1.040 | 1.001 | 1.034 | 1.110 | 1.044 |
| Pingshan | 1.282 | 1.268 | 1.054 | 1.075 | 0.590 | 0.748 | 0.666 | 0.955 |
| Guangming | 0.960 | 0.902 | 1.042 | 0.908 | 1.164 | 1.056 | 1.018 | 1.007 |
| Dapeng | 1.178 | 1.149 | 1.470 | 1.461 | 1.415 | 1.335 | 1.250 | 1.323 |
| Luohu | 0.814 | 0.773 | 0.695 | 0.755 | 0.757 | 0.793 | 0.766 | 0.764 |
| Mean | 1.062 | 1.053 | 1.047 | 1.005 | 0.967 | 1.025 | 1.007 | 1.024 |

**TABLE S2** SE-SBM analysis of CHCs in the Luohu Hospital Group from 2015 to 2021.

| **CHCs** | **2015** | **2016** | **2017** | **2018** | **2019** | **2020** | **2021** | **Mean** |
| --- | --- | --- | --- | --- | --- | --- | --- | --- |
| C1 | 1.129 | 1.074 | 0.542 | 0.509 | 1.036 | 1.013 | 1.002 | 0.901 |
| C2 | 1.275 | 1.104 | 1.161 | 1.011 | 1.019 | 1.008 | 1.014 | 1.085 |
| C3 | 0.533 | 0.454 | 0.409 | 0.508 | 0.559 | 0.520 | 0.586 | 0.510 |
| C4 | 1.257 | 1.183 | 0.283 | 1.193 | 1.346 | 1.454 | 1.860 | 1.225 |
| C5 | 1.102 | 1.684 | 1.570 | 1.367 | 1.312 | 1.256 | 1.300 | 1.370 |
| C6 | 1.210 | 1.075 | 1.002 | 1.008 | 1.062 | 1.049 | 0.748 | 1.022 |
| C7 | 0.645 | 0.592 | 0.534 | 0.496 | 0.548 | 1.016 | 0.639 | 0.638 |
| C8 | 1.244 | 1.351 | 1.140 | 1.041 | 1.028 | 1.008 | 1.001 | 1.116 |
| C9 | 1.003 | 1.020 | 1.070 | 1.133 | 0.616 | 1.029 | 1.149 | 1.003 |
| C10 | 1.710 | 1.422 | 1.726 | 1.532 | 1.321 | 1.316 | 1.433 | 1.494 |
| C11 | 1.167 | 1.114 | 1.254 | 1.453 | 1.486 | 1.210 | 1.187 | 1.267 |
| C12 | 1.003 | 1.112 | 1.152 | 1.011 | 1.133 | 1.191 | 1.207 | 1.115 |
| C13 | 1.032 | 0.656 | 1.009 | 1.090 | 0.537 | 1.044 | 0.830 | 0.885 |
| C14 | 1.135 | 1.273 | 1.129 | 1.121 | 0.650 | 1.013 | 1.010 | 1.047 |
| C15 | 1.061 | 1.098 | 0.444 | 1.135 | 1.042 | 1.022 | 0.776 | 0.940 |
| C16 | 0.349 | 0.325 | 1.010 | 1.200 | 1.324 | 1.207 | 1.083 | 0.928 |
| C17 | 1.155 | 1.105 | 1.053 | 1.070 | 1.115 | 1.019 | 1.065 | 1.083 |
| C18 | 1.034 | 1.083 | 1.014 | 1.042 | 1.169 | 1.219 | 1.166 | 1.104 |
| C19 | 1.158 | 1.184 | 1.092 | 1.018 | 1.111 | 1.086 | 1.098 | 1.107 |
| C20 | 1.076 | 1.015 | 1.135 | 1.014 | 1.056 | 1.187 | 1.111 | 1.085 |
| Mean | 1.064 | 1.046 | 0.987 | 1.048 | 1.023 | 1.093 | 1.063 | 1.046 |

**TABLE S3** Productivity change by CHCs during 2015–2021.

| **Years** | **EFFCH** | **TECHCH** | **PECH** | **SECH** | **TFPCH** |
| --- | --- | --- | --- | --- | --- |
| 2015-2016 | 0.983 | 1.206 | 0.965 | 1.018 | 1.168 |
| 2016-2017 | 0.954 | 0.983 | 0.994 | 0.952 | 0.917 |
| 2017-2018 | 1.119 | 0.944 | 1.098 | 1.006 | 1.004 |
| 2018-2019 | 1.011 | 1.159 | 0.971 | 1.034 | 1.140 |
| 2019-2020 | 1.071 | 0.837 | 1.083 | 0.985 | 0.859 |
| 2020-2021 | 0.978 | 1.422 | 0.959 | 1.024 | 1.383 |
| Mean | 1.020 | 1.092 | 1.012 | 1.003 | 1.078 |

**TABLE S4.** Tobit regression analysis results

| **Efficiency score** | **Coefficient** | **Std.Error** | **t** | ***P*>\|t\|** | **[95% conf. interval]** | |
| --- | --- | --- | --- | --- | --- | --- |
|  |  |  |  |  | **Lower** | **Upper** |
| **Regions** | | | | | | |
| PGDP | 0.0000 | 0.0000 | -0.2000 | 0.8450 | 0.0000 | 0.0000 |
| Population size | .0035 | 0.0040 | 0.8600 | 0.3910 | -0.0045 | 0.0114 |
| Health literacy level | -0.0024 | 0.0029 | -0.8400 | 0.4000 | -0.0081 | 0.0033 |
| _cons | 1.0144 | 0.0318 | 31.9000 | 0.0000 | 0.9515 | 1.0773 |
| Var (e. Efficiency score | 0.0902 | 0.1078 | - | - | 0.0712 | 0.1142 |
| Log likelihood | -30.2372 | - | - | - | - | - |
| **CHCs** | | | | | | |
| Percentage of GPs among health works | 1.2283 | 0.3335 | 3.6800 | 0.0000 | 0.5687 | 1.8879 |
| Percentage of intermediate and above staff in health works | 0.0000 | 0.0000 | 1.7700 | 0.0790 | 0.0000 | 0.0001 |
| Floor area | 0.0706 | 0.1135 | 0.6200 | 0.5350 | -0.1538 | 0.2950 |
| The number of home hospital beds | 0.0010 | 0.0004 | 2.5900 | 0.0110 | 0.0002 | 0.0018 |
| _cons | 0.4625 | 0.1457 | 3.1700 | 0.0020 | 0.1744 | 0.7506 |
| Var (e. Efficiency score | 0.0708 | 0.0085 | - | - | 0.0559 | 0.0897 |
| Log likelihood | -13.2890 | - | - | - | - | - |
